# Intraoperative use of gelatin in living donor liver transplantation and postoperative acute kidney injury

**DOI:** 10.1186/cc14437

**Published:** 2015-03-16

**Authors:** HK Atalan, B Gucyetmez, S Aslan, M Berktas, KY Polat

**Affiliations:** 1 Atasehir Memorial Hospital, Istanbul, Turkey; 2 International Hospital, Istanbul, Turkey; 3 Kappa Consulting, Istanbul, Turkey

## Introduction

The aim of our study is to investigate the effect of intraoperative use of gelatin in living donor liver transplantation on postoperative acute kidney injury (AKI). It has been demonstrated that ischemia and chloride-liberal fluid management cause AKI in liver transplantation [1]. Gelatin has minimal side effects on renal functions [2]; however, it might be a reason for postoperative AKI.

## Methods

A total of 154 liver transplantation patients were retrospectively evaluated between September 2011 and September 2013, and among these, 128 patients were included in the study. The patients who were under 18 years old, transplanted from cadaveric donors and needed preoperative renal replacement therapy were excluded. The patients were divided into two groups as GI (without gelatin administration) and GII (with gelatin administration). The patient's age, gender, actual body weight, diagnoses, MELD score, APACHE II score, duration of operation, total clamping time, noradrenalin infusion rate, amount of erythrocyte suspension, fresh frozen plasma (FFP) and thrombocyte suspension used, intraoperative fluid balance, intraoperative and total clamping diuresis, serum creatinine levels on the postoperative 1st, 2nd, 4th and 7th days, duration of mechanical ventilation, length of ICU and hospital stay, hospital and 1-year mortality rate were recorded. The changes in creatinine levels on the 1st, 2nd, 4th and 7th days were evaluated according to the KDIGO guideline for AKI [3].

## Results

In total, 128 patients were categorized as GI (58, 45%) or GII (70, 55%). Total clamping time, intraoperative diuresis, intraoperative crystalloid use, intraoperative fluid balance, operation bleeding, erythrocyte suspension, FFP and thrombocyte suspension use and postoperative lactate levels of GII were statistically significantly higher than GI (*P *< 0.001 for each). According to the KDIGO guideline, AKI in GII on the 1st, 2nd, 4th and 7th days (11.4%; 20%; 24.3%; 17.1%) was statistically significantly higher than GI (*P *< 0.001 for each).

## Conclusion

In patients who received gelatin, kidney dysfunction in the postoperative period was observed more frequently. Also in this group, total clamping time was longer and amount of blood products used during surgery was more than the other group. Which of these factors is associated with AKI has to be revealed with further studies.
